# Micromanufacturing Process of Complex 3D FeCo Core Microwindings for Magnetic Flux Modulation in Micromotors

**DOI:** 10.3390/mi17010115

**Published:** 2026-01-15

**Authors:** Efren Diez-Jimenez, Diego Lopez-Pascual, Gabriel Villalba-Alumbreros, Ignacio Valiente-Blanco, Miguel Fernandez-Munoz, Jesús del Olmo-Anguix, Oscar Manzano-Narro, Alexander Kanitz, Jan Hoppius, Jan Philipp

**Affiliations:** 1 Mechanical Engineering Area, Universidad de Alcalá, 28801 Alcalá de Henares, Spain; gabriel.villalba@uah.es (G.V.-A.); i.valiente@uah.es (I.V.-B.); miguel.fm@uah.es (M.F.-M.); j.olmo@uah.es (J.d.O.-A.); oscar.manzano@uah.es (O.M.-N.); 2 Electrical Engineering Area, Universidad de Alcalá, 28801 Alcalá de Henares, Spain; d.lopezp@uah.es; 3 Lidrotec GmbH, Universitätsstraße 150, 44801 Bochum, Germany; alexander.kanitz@lidrotec.com (A.K.); jan.hoppius@lidrotec.com (J.H.); jan-philipp.wessels@lidrotec.com (J.P.)

**Keywords:** MEMS, microcoils, micromanufacturing, laser micromachining

## Abstract

This work presents the design, fabrication, and characterization of a three-dimensional FeCo-based flux-modulator microwinding intended for integration into high-torque axial-flux Vernier micromotors. The proposed micromotor architecture modulates the stator magnetic flux using 12 magnetically isolated FeCo teeth interacting with an 11-pole permanent-magnet rotor. The design requires the manufacturing of complex three-dimensional micrometric parts, including three teeth and a cylindrical core. Such a complex design cannot be manufactured using conventional micromanufacturing lithography or 2D planar methods. The flux-modulator envelope dimensions are 250 μm outer diameter and 355 μm height. It is manufactured using a femtosecond laser-machining process that preserves factory-finished surfaces and minimizes heat-affected zones. In addition, this micrometric part has been wound using 20 μm diameter enamelled copper wire. A dedicated magnetic clamping fixture is developed to enable multilayer microwinding of the integrated core, producing a 17-turn inductor with a 60.6% fill factor—the highest reported for a manually wound ferromagnetic-core microcoil of this scale. Geometric and magnetic characterization validates the simulation model and demonstrates the field distribution inside the isolated core. The results establish a viable micromanufacturing workflow for complex 3D FeCo microwindings, supporting the development of next-generation high-performance MEMS micromotors.

## 1. Introduction

Microelectromechanical systems (MEMS) have emerged as a key technology within modern microelectronics. Recent progress in microscale electromagnetic devices, such as motors [1,2,3,4], clutches and brakes [5,6,7,8], magnetic micro-gears [9,10], vibrational energy harvesters and dampers [11], and other electromagnetic microsystems [12], is driven by their potential in high-precision optical instrumentation [13], data-storage electronics [14], optical positioning platforms [15], robotic inspection in confined environments [16], and micro-manipulation for actuator and transducer assembly [17].

Minimally invasive surgery represents a particularly impactful application field. Surgical intervention remains critical for managing numerous high-mortality conditions, with overall mortality for major procedures ranging from 0.5% to 5% and exceeding 30% in certain visceral operations [18]. Smaller surgical tools reduce procedural risk and recovery time, yet current tools have physical size limitations. For instance, catheter diameters typically range between 0.33 and 3 mm depending on clinical purpose [19]. Further miniaturization could significantly benefit interventions such as mitral valve repair [20], rotational atherectomy [21], neurosurgical tumour excision [22], cardiac arrhythmia ablation [23], and intracranial angioplasty [24]. Additionally, reduced instrument size may lower healthcare costs, as demonstrated in the transition from femoral to radial vascular access, which shortens recovery time and decreases resource use [25].

In macroscale actuation, torque amplification is usually achieved through mechanical gearheads [26]. However, implementing analogous gearing at the microscale introduces substantial complexity and part-count challenges [27,28]. As a result, existing micromotors exhibit comparatively low torque densities. For example, a stepping micromotor based on a ferrofluid bearing [29] and a flat epitaxial micromotor of 8 mm diameter incorporating helical coils [30] report torque densities around 0.025 kNm/m^3^, while another design reaches approximately 0.04 kNm/m^3^ [31]. An improved microactuator of 2 mm diameter achieves up to 0.2 kNm/m^3^ [32], yet this remains one order of magnitude lower than the 0.6–8 kNm/m^3^ typical of highly efficient macroscale motors.

Magnetic gearboxes are therefore of strong interest, offering torque multiplication without mechanical contact. By using controlled interactions between permanent magnets, these systems reproduce the torque-transmission behaviour of conventional gears without the associated wear, heat generation, or lubrication requirements. A key configuration is the concentric flux-modulated magnetic gear, consisting of two permanent-magnet rings and an intermediate flux-modulating structure composed of magnetically isolated ferromagnetic teeth. Rotation of one element induces modulated magnetic flux, generating a corresponding rotational response in the paired rotor.

At the microscale, the design and fabrication of the flux modulator are particularly critical, as its geometry directly determines the magnetic coupling efficiency and the feasibility of torque transmission. Complex three-dimensional, high-aspect-ratio components fabricated from ferromagnetic alloys such as FeCo or NiFe are required for advanced electromagnetic applications. Small-scale bulk FeCo parts have been successfully produced via micro-fast sintering at relatively low processing temperatures, yielding grain sizes of 5–6 μm and exhibiting a high degree of densification [33]. Further advancements have been reported for electron-beam welding of ultra-thin FeCo-V foils, achieving notable microstructural and functional performance [34]. Additive manufacturing has also emerged as a promising route for generating high-aspect-ratio features in soft magnetic composites [35]. In parallel, powder injection moulding has been developed to fabricate fully dense components without the need for vanadium alloying, offering improved quality compared to conventionally sintered parts [36]. Despite these developments, the aforementioned techniques share significant limitations, particularly insufficient structural integrity and the inability to reliably produce micrometre-scale features with complex geometries. In contrast, state-of-the-art micromachining technologies enable the creation of intricate two- and three-dimensional geometries through precision material removal in high-magnetic-performance FeCo workpieces [37]. Among the most accurate approaches are Pulse Laser Ablation (PLA) [38], Electrical Discharge Machining (EDM), Focused Ion Beam (FIB), and chip-based micromachining processes. PLA facilitates high-precision fabrication of complex small-scale planar features and offers favourable scalability [39]; however, its applicability is constrained by material surface quality, optical absorption characteristics, and the potential degradation of magnetic properties due to elevated processing temperatures.

In this work, we describe an innovative method for micromanufacturing and winding of 3D FeCo microparts. A specialized femtosecond laser-based machining method is used, which minimizes the heat-affected zone, thereby preserving the intrinsic magnetic properties of the material while achieving excellent geometrical fidelity. Geometric and magnetic characterization of the results show good agreement with mechanical design and simulated electromagnetic behaviour.

## 2. MEMS and Micromotor Design

**Figure 1:**
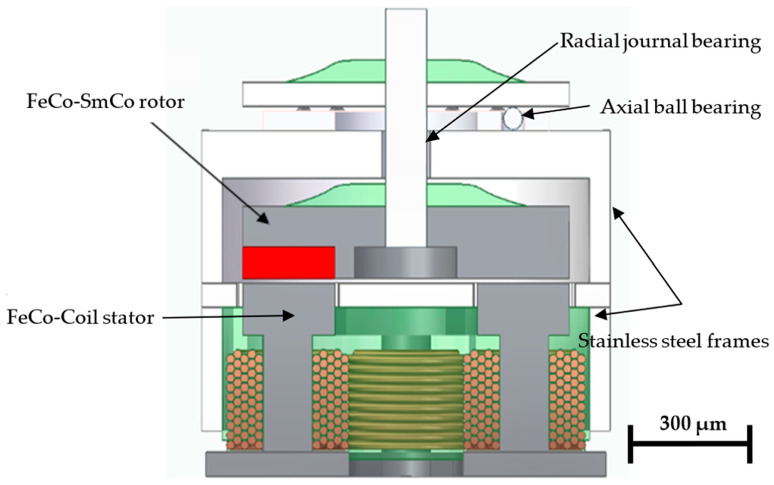
Mechanical and magnetic components of the high-performance design of the proposed micromotor. Motor outer diameter (OD) = 1000 μm, motor height = 1000 μm. Air gap = 10 μm.

Multipolar arrangements enable the development of compact micromachines with enhanced torque density and efficiency. In this context, a new axial-flux Vernier stepper micromotor is proposed to increase the torque density achievable in MEMS-based rotary actuation. The topology incorporates four stator iron cores driven in two electrical phases, together with a magnetic flux modulator designed to amplify torque transmission while significantly reducing cogging torque. The rotor consists of 11 permanent-magnet dipoles, while the flux modulator comprises 12 magnetically isolated soft-ferromagnetic teeth, producing a Vernier magnetic gearing effect with an effective 1:11 torque transformation ratio. This gearing mechanism is essential for mitigating scale-induced detent torque. The mechanical assembly further includes an axial ball micro-bearing [40] and a planar radial bearing to maintain rotor alignment. Non-magnetic structural components are manufactured in titanium or non-magnetic stainless steel (e.g., AISI-316). The overall micromotor design is shown in Figure 1.

**Figure 2:**
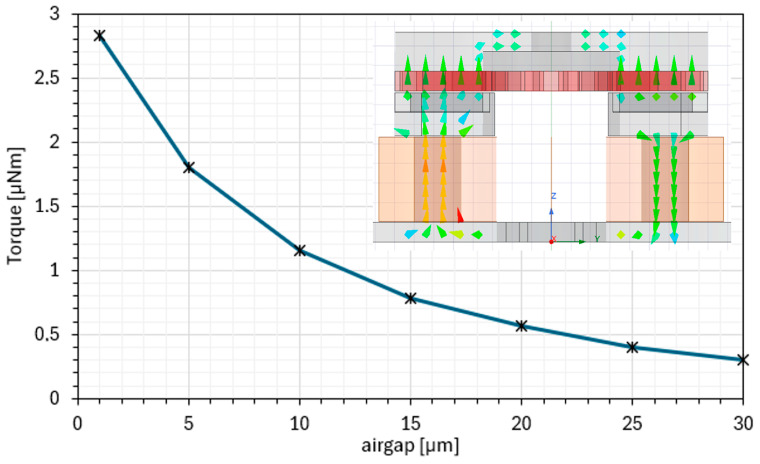
Influence of the airgap between rotor and stator on the resulting torque.

This work focuses on the FeCo-Coil stator manufacturing, ensuring compatibility with the selected processes and available magnetic materials. Machined solid ferromagnetic materials are used instead of laminations or additively manufactured alloys due to their superior magnetic performance, mechanical integrity, and surface finish quality. Surface quality is critical, as the motor requires an extremely small airgap to maintain high magnetic flux density. Electromagnetic simulations indicate that airgap directly affects magnetic circuit reluctance: halving the airgap (e.g., to 5 μm) increases torque by approximately 55%, while enlarging it to 15 μm reduces torque by around 31% (See Figure 2). This makes geometric tolerances and assembly precision primary determinants of performance.

**Figure 3:**
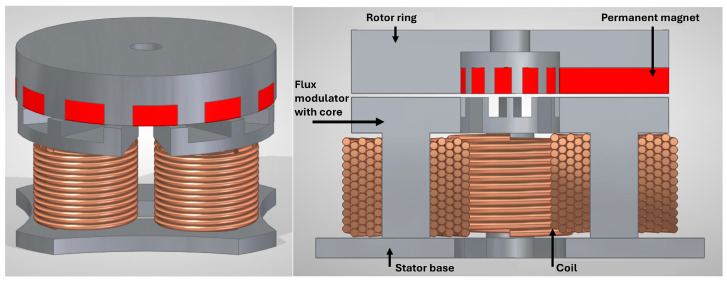
Design of the rotor and stator configuration for the Vernier motor.

Because a monolithic 3D stator core cannot be manufactured or wound with the required precision, the stator must be segmented. However, segmentation introduces local increases in magnetic reluctance at the interfaces, reducing torque. Several segmentation strategies were evaluated and quantified. The solution selected divides the stator into five components: four flux-modulator-plus-core units and one ferromagnetic base (see Figure 3). This configuration balances electromagnetic efficiency, manufacturability, coil winding feasibility, and assembly alignment reliability.

**Figure 4:**
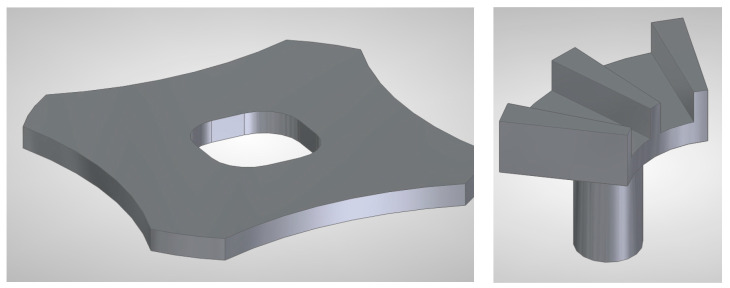
Ferromagnetic parts that form the stator. Baseplate (**left**) and flux modulator with core (**right**).

The modulators and cores are fabricated from 355 μm Hiperco 50 FeCo alloy, allowing the upper airgap-forming surfaces to retain supplier-grade surface finish. The final stator parts’ geometries are shown in Figure 4. In order to assemble the stator, once the four flux modulators are manufactured and winded, they would be adhered onto the ferromagnetic base using a low-viscosity epoxy adhesive.

The rotor is produced from 150 μm Vacodur 49, forming a ferromagnetic ring with recesses for permanent-magnet insertion and a 100 μm central bore for shaft coupling. The rotor manufacturing has been presented in [41]. Magnet integration quality is critical; final torque output depends not only on magnet grade but also on assembly precision and magnetic interface continuity.

## 3. Microfabrication of the Flux Modulator

In this work, the FeCo-2V alloy is selected to manufacture the ferromagnetic parts. FeCo alloys are typically characterized by low ductility and limited workability. To improve machinability and mechanical rigidity, vanadium is commonly added to the alloy composition. This addition leads to a slight reduction in magnetic performance, including lower magnetic permeability and increased coercivity [42]. The nominal composition is of 49% Co, 49% Fe, and 2% V, and offers high saturation magnetisation together with greater mechanical strength than conventional soft magnetic alloys. This material is supplied in cold-rolled sheet form with thicknesses between 55 µm and 355 µm, and presents typical mechanical properties including a yield strength of 1150–1270 MPa, tensile strength of 1230–1310 MPa, Poisson’s ratio of ~0.29, Young’s modulus of 200–206 GPa, hardness of 342–380 HV, density of 8.12 g/cm^3^, and a thermal expansion coefficient of 8.9–9.7 × 10^−6^ K^−1^ (20–200 °C)

**Figure 5:**
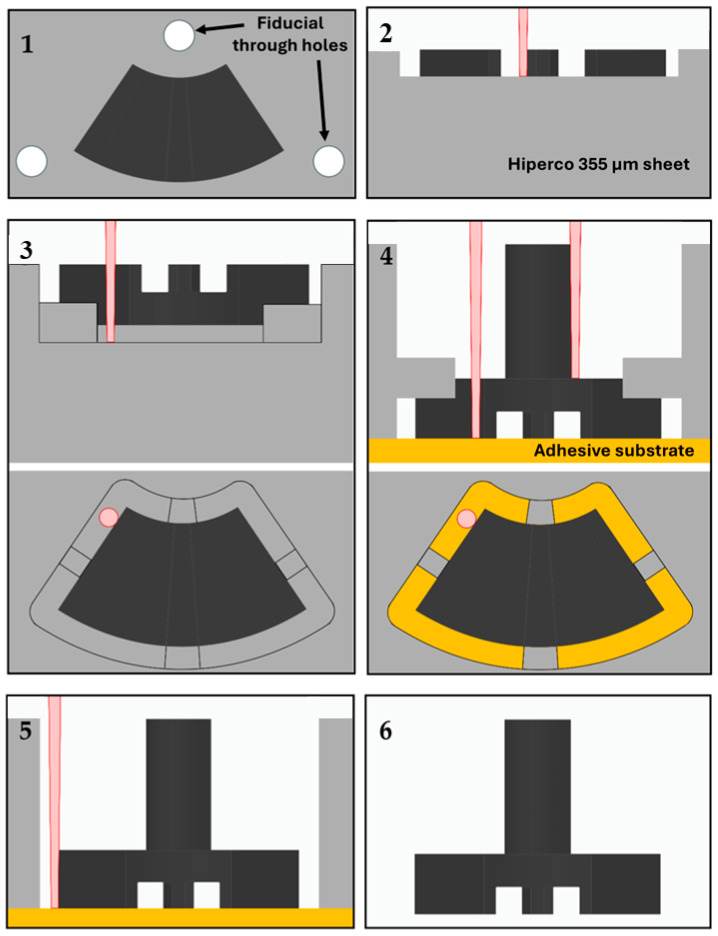
Full manufacturing process diagram of the flux modulator with core by laser machining.

This part is manufactured by special femtosecond laser machining of the raw Hiperco 50 (FeCo-2V) sheet from both sides, since the precision of the conventional micro machining techniques used for the manufacturing of the base plate achieve lesser tolerances. This process will also ensure that the top surface of the teeth and the bottom surface of the core remain factory finished. This is crucial for maintaining the minimal stator–rotor airgap and for accurate assembly with the stator base. The tooth height (50 µm) and the perpendicularity of the tooth faces are the primary dimensional tolerances required for correct alignment with the axial retainer in the Vernier actuator. Some of the more critical measurements are the core diameter (120 ± 10 µm) and internal and external radius of the flux modulator (180 ± 10 µm and 400 ± 10 µm, respectively) The manufacturing plan and machining sequence starts with the machining of four fiducial holes for precise positioning after turning the FeCo sheet to machine the lower face. These holes are positioned at a distance of 350 µm from the centre of the flux modulator core. The machining process of the upper face then consists of cutting the tooth gaps and shaping the outer profile while leaving temporary support tabs. Then the sheet is turned, and lower face is machined while the part is fixed to an adhesive substrate where the yoke geometry is defined, the final profile is completed, and the support tabs are then removed to release the piece. This whole process is shown in Figure 5, where the already processed part of the flux modulator with core is highlighted in a darker colour.

A slight misalignment between the core and flux modulator geometry may occur during this two-sided process; however, this is not critical to functionality.

The laser milling system employed in this study utilizes a commercial ultrafast laser source (TRUMPF TrueMicro 2030, Madrid, Spain) operating at a wavelength of 1030 nm, with a pulse duration of 400 fs, a maximum pulse energy of 100 µJ, and an average power of 20 W. The workpiece is mounted on a liquid-cooled chuck. The cutting speed is set to 30 µm/s, and water is used as the processing medium. The water layer thickness, typically approximately 10 mm, was selected in accordance with the recommendations in [43]. Proper alignment between the focusing lens and the liquid layer height is essential to ensure optimal beam focusing on the workpiece surface. The water remains at room temperature and provides ultrafast cooling on the nanosecond timescale, as previously demonstrated both theoretically and experimentally in [44,45].

The laser milling system comprises the laser source, beam-delivery mirrors, shutter, aperture, electronic power attenuator, a high-precision galvanometer scanner, a focusing lens, and high-accuracy XYZ linear stages. The laser beam spot, with a radius of approximately 8 µm, is produced by a 70 mm focal-length lens. The combined galvanometer scanner and linear translation stages enable precise positioning in all three spatial directions (X, Y, and Z) with a positional accuracy of 0.1 µm. The principal challenge of the process lies in machining microparts of this scale without inducing structural damage or fracture, particularly given the inherent fragility of the components. Achieving high-quality surfaces on both machined faces is essential. Furthermore, the absence of debris, fractures, or burrs is critical, as surface integrity and dimensional precision constitute fundamental requirements for subsequent integration and assembly stages.

**Figure 6:**
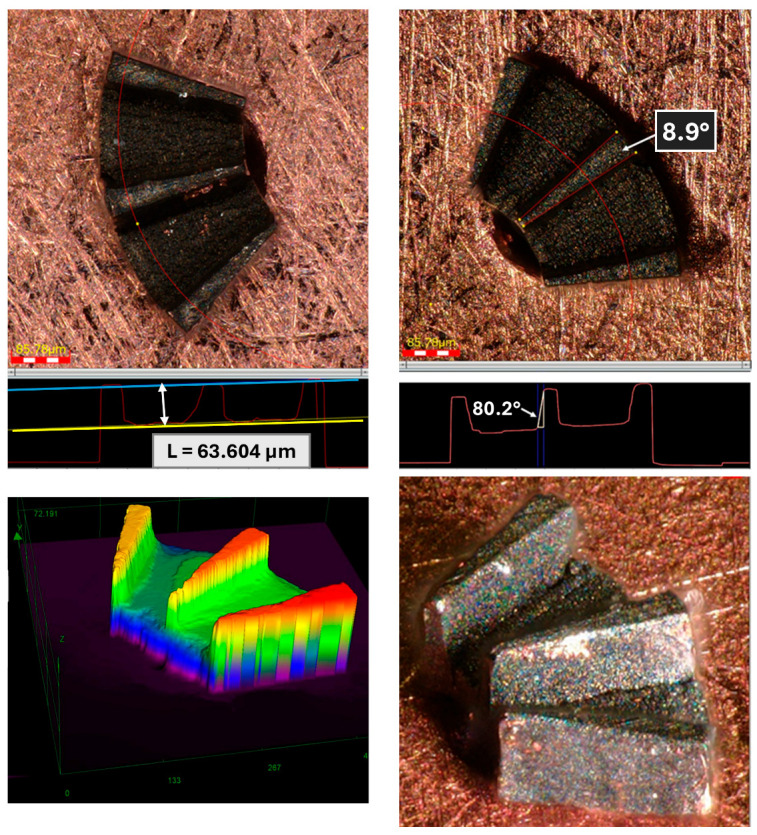
Measurements of the upper face of the flux modulator with core (**top**) and 3D microscope images (**bottom**). Cylindrical core is not shown as it is inserted into a hole to hold the part horizontally.

Figure 6 presents the measured dimensions of the flux-modulator teeth. The angle of the teeth ranges between 8° and 9° across the examined samples, with a general wall inclination of approximately 10° caused by the laser-machining process. This taper decreases as the machining depth increases. The measured tooth depth is 60 ± 5 µm, slightly exceeding the 50 µm specified in the design. Although these slight deviations do not affect the actuator’s performance, the corresponding models must be updated to ensure proper fitting between the teeth and the axial retainer within the established tolerances. The resulting component exhibits high manufacturing quality; the horizontal surfaces are flat and uniform, and the edges and walls are sharply defined, ensuring accurate alignment with the axial retainer during assembly. It is important to note that the top and bottom surfaces have not been affected by the machining process, and that the angle observed in the microscope measurements is caused by the difficult positioning of the part for measurement given its size and geometry. Moreover, no heat-affected zones are observed.

**Figure 7:**
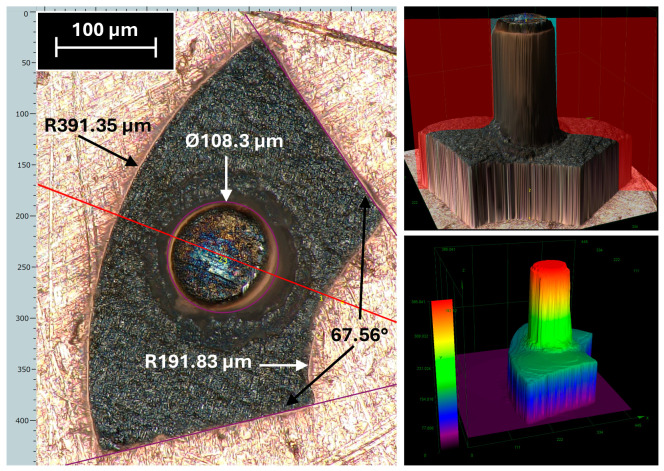
Measurements of the lower face of the flux modulator with core (**left**) and 3D microscope images (**right**).

Figure 7 presents the measurements corresponding to the shape of the flux modulator and the cylindrical core viewed from below. Once again, the surface quality is highly satisfactory, particularly regarding the perpendicularity of the core walls, whose thickness ranges between 110 and 120 µm, depending on the measurement height. For the actuator design, slight deviations in core cylindricity or cross-sectional accuracy are non-critical and therefore acceptable. The heights of the core and the modulator match the design specifications. A notable advantage of this manufacturing process is that the total height of the component remains consistent across all samples, facilitating accurate alignment during assembly.

## 4. Flux Modulator Winding and Assembly

A second step in the construction is the winding process of the stator magnetic core. First, the material that will act as the rigid core for the winding is positioned. In the case of the flux modulator, due to its small size, the part cannot be clamped directly by the core section because doing so would reduce the available winding length. Similarly, clamping via the modulator head body is not feasible, as the pressure exerted by the chuck would deform the tooth geometry. Therefore, an auxiliary holding mechanism is required. Since the part is magnetic, magnetic holding solutions are considered.

**Figure 8:**
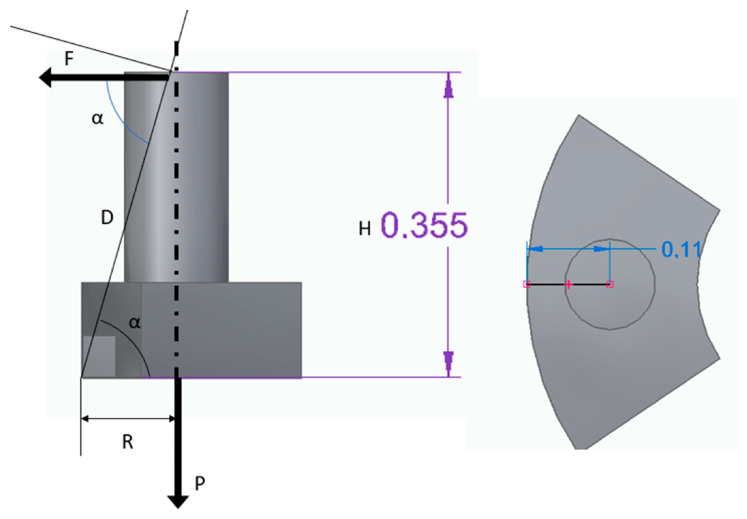
Force diagram that affects the flux modulator with the core while winding.

Applying force equilibrium to the diagram in Figure 8, the attractive force “P” acting on the component must be equal to or greater than 3.22 times the tensile force “F” applied by the wire tensioning system. Considering a minimum tension of 25 mN, the minimum required attractive force to prevent tipping is 80 mN. Additionally, to prevent sliding on the magnet and assuming a typical static friction coefficient of 0.74 (steel–steel), the minimum required attractive force is 34 mN. Therefore, tipping is the limiting condition.

**Figure 9:**
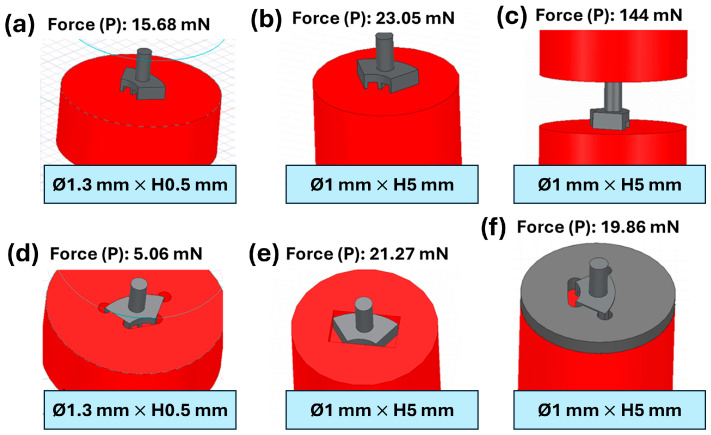
Different types of magnetic grips based on magnets with Sm_2_Co_17_ properties and the axial force they exert on the flux modulator with core.

To achieve the required attractive force, several magnetic holding systems using axially magnetized permanent magnets were designed. Figure 9 shows different possible holding configurations. Option (c) provides sufficient holding force but requires an auxiliary axial sliding support system for the second magnet, increasing mechanical complexity and alignment requirements. The most practical solution is configuration (e), which uses a single magnet and a machined cavity that prevents tipping and sliding, while the magnetic attraction maintains the component in place during machining. Solutions such as (f) are discarded because they require additional bonding steps, and smaller magnets are excluded due to handling difficulties.

**Figure 10:**
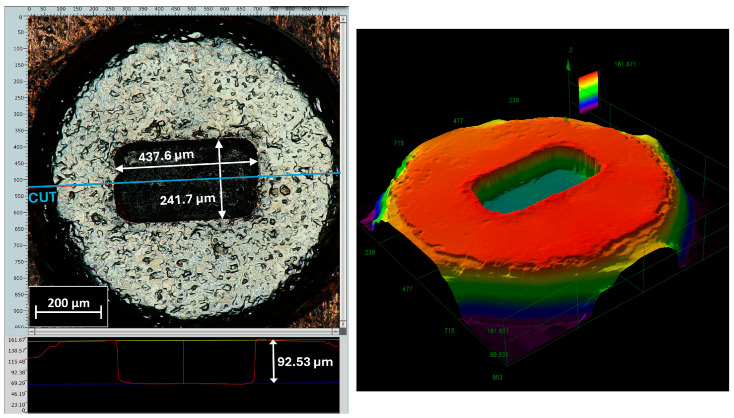
Magnetic grip manufactured by milling; measurements (**left**) and three-dimensional representation of the machined cavity (**right**).

Figure 10 shows the machined clamping magnet, demonstrating high surface quality with smooth internal surfaces and straight walls that prevent part displacement. The cavity depth is slightly smaller than the height of the modulator, allowing the wire to return and cover the full available winding length.

The wire is guided manually at low speed. Automatic guiding is unnecessary, as the adhesive coating naturally promotes layer-to-layer alignment. The calibrated grid ensures proper spacing, and visual inspection verifies packing uniformity.

**Figure 11:**
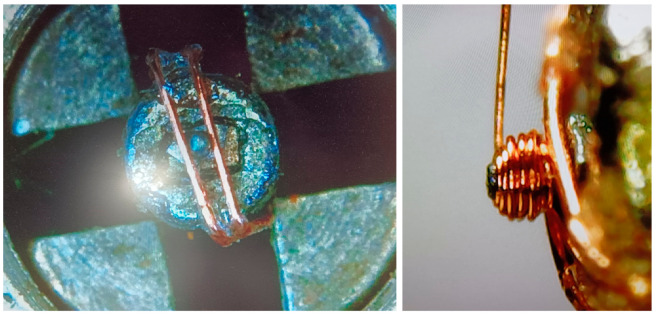
Definitive solution for the winding of the flux modulator with core.

After several attempts, the resulting coils exhibited a very low fill factor due to the difficulty of winding the wire under such low tension, which is limited by the magnetic attraction force. To prevent the part from being ejected from the magnetic chuck in the event of localized increases in cable tension during winding, two additional wires were incorporated into the assembly. This enables winding at a consistently higher tension level and, therefore, improves fill factor. Although this results in a slight reduction in the usable core length, it enables a significantly higher fill factor in the winding and ensures a greater number of turns compared with previous attempts without the fixing wires. The wires do not align the part radially nor axially. Magnetic clamping was essential to align and keep the piece in the right position during winding. This solution is displayed in Figure 11.

After finishing, the wire is cut, leaving extra length for later integration, and the wound core is removed and stored.

**Figure 12:**
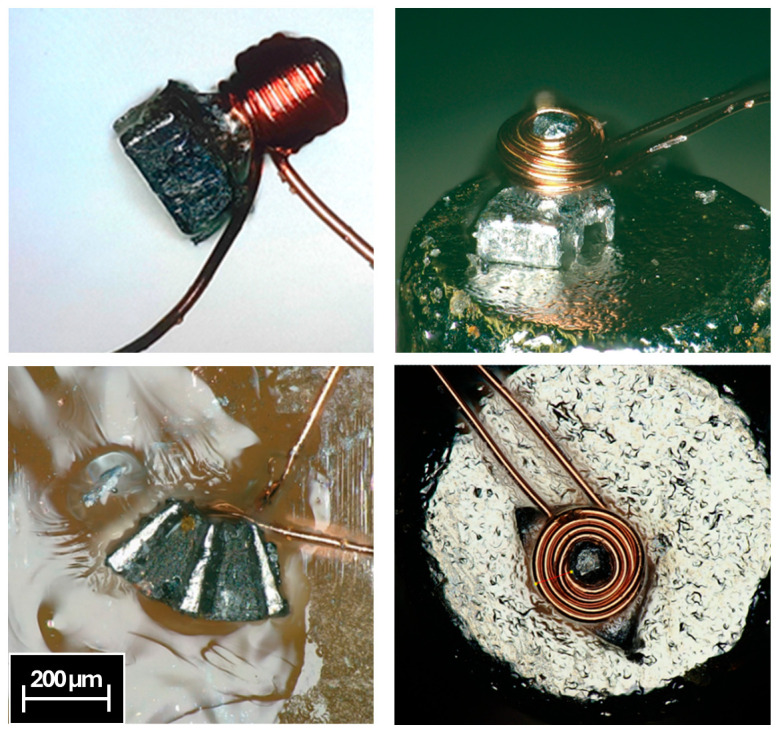
Completed flux modulator with coil.

For the flux modulator core, the resulting winding has a diameter of 285 µm, a copper thickness of 63 µm, and a length of 140 µm. It consists of four layers of 20 µm wire, with approximately four turns per layer, totalling 17 turns and yielding a fill factor of 60.6%. Figure 12 presents several views of the wound component. The winding process is more complex due to limited working length; however, this constitutes the smallest multilayer ferromagnetic-core inductor fabricated to date, with fill factor exceeding that of lithographically produced micro-inductors.

## 5. Magnetic Characterization

**Figure 13:**
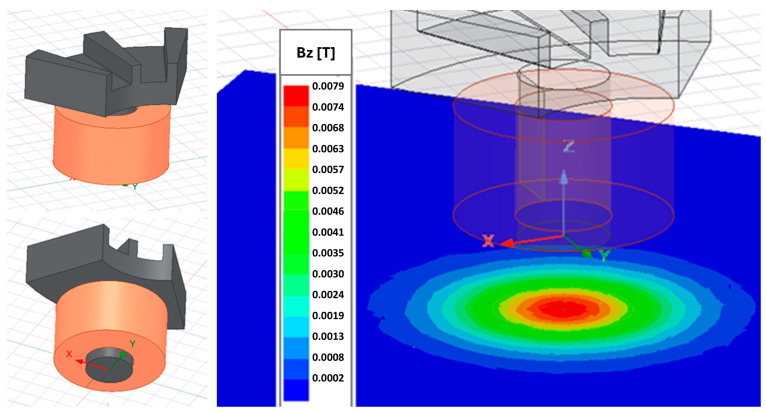
Magnetic flux modulator with wound core; simulation model and results.

The resulting winding micropart has been magnetically characterized and compared with a simulation model. The simulation model of the wound modulator is shown in Figure 13. The winding is replaced by a copper cylinder with equivalent width and length to simplify the simulation.

A current of 0.15 A is applied experimentally, corresponding to a current density of 477 A/mm^2^ on the cable, delivered intermittently to prevent thermal damage. In the simulation, the total equivalent current for the 17 turns of the coil (2.55 A) is applied.

**Figure 14:**
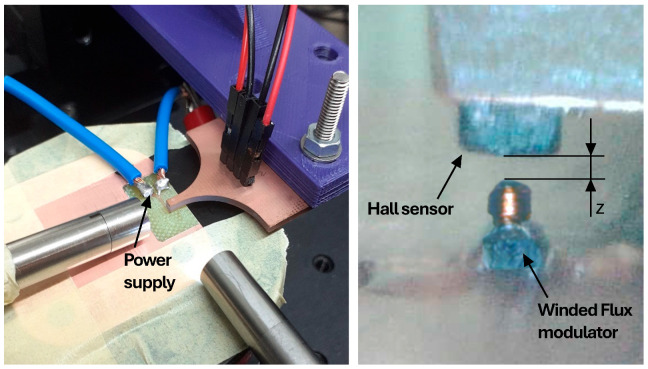
Experimental setup for the magnetic flux modulator with wound core.

Magnetic measurements have been performed using a micro hall sensor (HG07-11, AKM company, Tokyo, Japan) mounted on a high precision 3-axis stage, which allows to precisely move the hall sensor relative to the flux modulator. This setup is shown in Figure 14.

**Figure 15:**
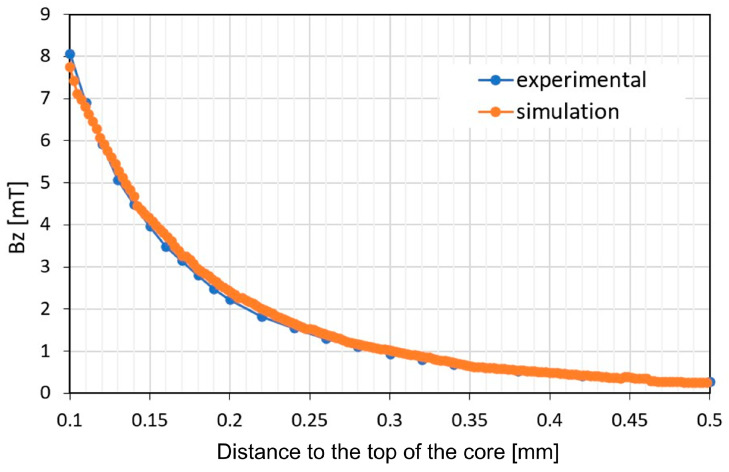
Experimental magnetic field measurements with increasing distances from the top of the core, and comparison with simulation results.

The core of the flux modulator behaves as a dipole, generating a maximum field at its centre (see Figure 13). Once the peak magnetic field position is determined in the X-Y plane, the axial field is measured along the core axis with increasing distance to the top of the flux modulator core (z). The comparison shown in Figure 15 validates the model for both the winding and the laser-machined core.

Despite the high current density, the axial field inside the core remains low because the isolated core presents high magnetic reluctance, causing field lines to diverge quickly from one end of the winding to the other. This explains the absence of significant axial field in the modulator region.

The good agreement between simulation and experimental results permits the claim that heat-affected zone is residual, assuring that magnetic properties of the FeCo are the same than the original raw material.

## 6. Conclusions

This work demonstrates a complete micromanufacturing workflow for producing complex three-dimensional FeCo-2V flux-modulator microwindings suitable for axial-flux Vernier micromotors. The two-sided femtosecond laser-machining process applied to FeCo alloys enables the fabrication of magnetically isolated teeth and integrated cores with excellent dimensional accuracy, surface integrity, and preserved factory-grade finish—key factors for achieving the ultra-small airgaps required for high-torque density. Metrological inspection confirms consistent tooth geometry, minimal taper, and precise component height, ensuring reliable alignment within the actuator.

A novel magnetic clamping strategy was developed to enable multilayer winding on the microscale core, overcoming the limitations of direct mechanical fixation. The resulting coils achieve 17 turns and a 60.6% fill factor, representing one of the highest packing efficiencies reported for hand-wound ferromagnetic-core microinductors of comparable size. Magnetic characterization validates the finite-element model and confirms the expected field distribution inside the isolated core, despite the inherently high magnetic reluctance of the geometry.

Overall, the results demonstrate that high-quality 3D FeCo microwindings can be manufactured reproducibly with standard sheet alloys, providing a viable pathway for integrating flux-modulator stators into next-generation MEMS micromotors requiring enhanced torque performance and stringent geometric precision.

## Data Availability

The data presented in this study are available on request from the corresponding author.

## References

[1] Cao T., Hu T., Zhao Y. (2020). Research status and development trend of MEMS switches: A review. Micromachines.

[2] Serrano-Tellez J., Romera-Juarez F., González-De-María D., Lamensans M., Argelaguet-Vilaseca H., Pérez-Díaz J.-L., Sánchez-Casarrubios J., Díez-Jiménez E., Valiente-Blanco I. Experience on a cryogenic linear mechanism based on superconducting levitation. Proceedings of the Conference on Modern Technologies in Space-and Ground-Based Telescopes and Instrumentation II.

[3] Wu S., Zuo S., Wu X., Lin F., Shen J. (2016). Magnet modification to reduce pulsating torque for axial flux permanent magnet synchronous machines. Appl. Comput. Electromagn. Soc. J..

[4] Rezaeealam B., Rezaee-Alam F. (2017). Optimization of permanent magnet synchronous motors using conformal mappings. Appl. Comput. Electromagn. Soc. J..

[5] Rizzo R., Musolino A., Bucchi F., Forte P., Frendo F. (2015). A multi-gap magnetorheological clutch with permanent magnet. Smart Mater. Struct..

[6] Rizzo R. (2017). An innovative multi-gap clutch based on magneto-rheological fluids and electrodynamic effects: Magnetic design and experimental characterization. Smart Mater. Struct..

[7] Rizzo R., Musolino A., Lai H.C. (2017). An Electrodynamic/Magnetorheological Clutch Powered by Permanent Magnets. IEEE Trans. Magn..

[8] Hua D., Liu X., Li Z., Fracz P., Hnydiuk-Stefan A., Li Z. (2021). A Review on Structural Configurations of Magnetorheological Fluid Based Devices Reported in 2018–2020. Front. Mater..

[9] Muñoz-Martínez M., Diez-Jimenez E., Gómez-García M.J., Rizzo R., Musolino A. (2019). Torque and bearing reaction forces simulation of micro-magnetic gears. Appl. Comput. Electromagn. Soc. J..

[10] Ruiz-Ponce G., Arjona M.A., Hernandez C., Escarela-Perez R. (2023). A Review of Magnetic Gear Technologies Used in Mechanical Power Transmission. Energies.

[11] Sodano H.A., Bae J.S. (2004). Eddy current damping in structures. Shock Vib. Dig..

[12] Barmada S., Musolino A., Rizzo R. (2006). Equivalent network approach for the simulation of MEMS devices. Appl. Comput. Electromagn. Soc. J..

[13] Baltzer M., Obermeier E. A micro shutter for applications in optical and thermal detectors. Proceedings of the International Solid State Sensors and Actuators Conference (Transducers’ 97).

[14] Mourlas N.J., Stark K.C., Mehregany M., Phillips S.M. Exploring polysilicon micromotors for data storage micro disks. Proceedings of the Ninth International Workshop on Micro Electromechanical Systems.

[15] Bodnicki M., Wierciak J., Credo W., Bagiński K., Wawrzyniuk L. (2018). Electromagnetic angular positioner based on DC micromotor. MATEC Web Conf..

[16] Suzumori K., Miyagawa T., Kimura M., Hasegawa Y. (1999). Micro inspection robot for 1-in pipes. IEEE/ASME Trans. Mechatron..

[17] Thielicke E., Obermeier E. (2000). Microactuators and their technologies. Mechatronics.

[18] Baum P., Diers J., Lichthardt S., Kastner C., Schlegel N., Germer C.-T., Wiegering A. (2019). Mortality and Complications Following Visceral Surgery. Dtsch. Arztebl. Int..

[19] Meena K.V., Sankar A.R. (2021). Biomedical Catheters with Integrated Miniature Piezoresistive Pressure Sensors: A Review. IEEE Sens. J..

[20] Otto C.M. (2003). Timing of surgery in mitral regurgitation. Heart.

[21] Chiang M.H., Yi H.-T., Tsao C.-R., Chang W.-C., Su C.-S., Liu T.-J., Liang K.-W., Ting C.-T., Lee W.-L. (2013). Rotablation in the treatment of high-risk patients with heavily calcified left-main coronary lesions. J. Geriatr. Cardiol..

[22] Badie B., Brooks N., Souweidane M.M. (2004). Endoscopic and minimally invasive microsurgical approaches for treating brain tumor patients. J. Neurooncol..

[23] Miyamoto K., Tsuchihashi K., Uno K., Shimoshige S., Yoshioka N., Doi A., Nakata T., Shimamoto K. (2001). Studies on the prevalence of complicated atrial arrhythmias, flutter, and fibrillation in patients with reciprocating supraventricular tachycardia before and after successful catheter ablation. PACE—Pacing Clin. Electrophysiol..

[24] Abou-Chebl A., Krieger D.W., Bajzer C.T., Yadav J.S. (2006). Intracranial angioplasty and stenting in the awake patient. J. Neuroimaging.

[25] Hussain A., Kaul U. (2012). RadIal Vs FemorAL (RIVAL) trial for coronary angiography and intervention in patients with acute coronary syndromes. Indian Heart J..

[26] Perez-Diaz J.L., Diez-Jimenez E., Valiente-Blanco I., Cristache C., Alvarez-Valenzuela M.-A., Sanchez-Garcia-Casarrubios J., Ferdeghini C., Canepa F., Hornig W., Carbone G. (2015). Performance of Magnetic-Superconductor Non-contact Harmonic Drive for Cryogenic space applications. Machines.

[27] Diez-Jimenez E., Sanchez-Montero R., Martinez-Muñoz M. (2017). Towards miniaturization of magnetic gears: Torque performance assessment. Micromachines.

[28] Lu Z., Huang B., Zhang Q., Lu X. (2018). Experimental and analytical study on vibration control effects of eddy-current tuned mass dampers under seismic excitations. J. Sound Vib..

[29] Jayhooni S.M.H., Assadsangabi B., Takahata K. (2018). A stepping micromotor based on ferrofluid bearing for side-viewing microendoscope applications. Sens. Actuators A Phys..

[30] Waldschik A., Feldmann M., Seidemann V., Büttgenbach S., Waldschik A., Feldmann M., Seidemann V., Büttgenbach S. (2011). Development and Fabrication of Electromagnetic Microactuators. Design and Manufacturing of Active Microsystems.

[31] Koser H., Lang J.H. (2006). Magnetic Induction Micromachine—Part II: Fabrication and Testing Florent. J. Microelectromech. Syst..

[32] Kim J.H., Jung I.S., Sung H.G. Design and manufacturing of ultra small actuator. Proceedings of the 2006 IEEE International Conference on Mechatronics, ICM.

[33] Zhou B., Yang Y., Qin Y., Yang G., Wu M. (2022). Fabrication of equiatomic FeCo alloy parts with high magnetic properties by fields activated sintering. Manuf. Rev..

[34] Mostaan H., Shamanian M., Safari M. (2016). Process analysis and optimization for fracture stress of electron beam welded ultra-thin FeCo-V foils. Int. J. Adv. Manuf. Technol..

[35] Chang T.W., Liao K.W., Lin C.C., Tsai M.C., Cheng C.W. (2021). Predicting magnetic characteristics of additive manufactured soft magnetic composites by machine learning. Int. J. Adv. Manuf. Technol..

[36] Silva A., Lozano J.A., Machado R., Escobar J.A., Wendhausen P.A.P. (2008). Study of soft magnetic iron cobalt based alloys processed by powder injection molding. J. Magn. Magn. Mater..

[37] Villalba-Alumbreros G., Lopez-Camara E., Martínez-Gómez J., Cobreces S., Valiente-Blanco I., Diez-Jimenez E. (2023). Experimental study of micromilling process and deburring electropolishing process on FeCo-based soft magnetic alloys. Int. J. Adv. Manuf. Technol..

[38] Deng D., Wan W., Huang Q., Huang X., Zhou W. (2016). Investigations on laser micromilling of circular micro pin fins for heat sink cooling systems. Int. J. Adv. Manuf. Technol..

[39] Raciukaitis G. (2021). Ultra-Short Pulse Lasers for Microfabrication: A Review. IEEE J. Sel. Top. Quantum Electron..

[40] Michałowski M., Fernandez-munoz M., Samsel M.J., Cha A. (2023). Tribological Characterization of Micro Ball Bearings with and without Solid-State Lubrication. Micromachines.

[41] Diez-Jimenez E., Bollero A., Valiente-Blanco I., Palmero E.M., Fernandez-Munoz M., Lopez-Pascual D., Villalba-Alumbreros G. (2024). Integration of Sm2Co17 Micromagnets in a Ferromagnetic Multipolar Microrotor to Enhance MEMS and Micromotor Performance. Micromachines.

[42] Yu J., Yingchun Z., Jing J. Nonlinear MPC for attitude system of miniature satellite using multiple MEMS actuators. Proceedings of the 10th World Congress on Intelligent Control and Automation.

[43] Menéndez-Manjón A., Wagener P., Barcikowski S. (2011). Transfer-matrix method for efficient ablation by pulsed laser ablation and nanoparticle generation in liquids. J. Phys. Chem. C.

[44] Shih C.Y., Streubel R., Heberle J., Letzel A., Shugaev M.V., Wu C., Schmidt M., Gökce B., Barcikowski S., Zhigilei L.V. (2018). Two mechanisms of nanoparticle generation in picosecond laser ablation in liquids: The origin of the bimodal size distribution. Nanoscale.

[45] Kanitz A., Hoppius J.S., Fiebrandt M., Awakowicz P., Esen C., Ostendorf A., Gurevich E.L. (2017). Impact of liquid environment on femtosecond laser ablation. Appl. Phys. A Mater. Sci. Process..

